# Mobilization and Manipulation of the Cervical Spine in Patients with Cervicogenic Headache: Any Scientific Evidence?

**DOI:** 10.3389/fneur.2016.00040

**Published:** 2016-03-21

**Authors:** Jodan D. Garcia, Stephen Arnold, Kylie Tetley, Kiel Voight, Rachael Anne Frank

**Affiliations:** ^1^Department of Physical Therapy, Georgia State University, Atlanta, GA, USA

**Keywords:** cervicogenic headache, mobilization, manipulation, headache duration, headache frequency

## Abstract

Cervical mobilization and manipulation are frequently used to treat patients diagnosed with cervicogenic headache (CEH); however, there is conflicting evidence on the efficacy of these manual therapy techniques. The purpose of this review is to investigate the effects of cervical mobilization and manipulation on pain intensity and headache frequency, compared to traditional physical therapy interventions in patients diagnosed with CEH. A total of 66 relevant studies were originally identified through a review of the literature, and the 25 most suitable articles were fully evaluated *via* a careful review of the text. Ultimately, 10 studies met the inclusion criteria: (1) randomized controlled trial (RCT) or open RCT; the study contained at least two separate groups of subjects that were randomly assigned either to a cervical spine mobilization or manipulation or a group that served as a comparison; (2) subjects must have had a diagnosis of CEH; (3) the treatment group received either spinal mobilization or spinal manipulation, while the control group received another physical therapy intervention or placebo control; and (4) the study included headache pain and frequency as outcome measurements. Seven of the 10 studies had statistically significant findings that subjects who received mobilization or manipulation interventions experienced improved outcomes or reported fewer symptoms than control subjects. These results suggest that mobilization or manipulation of the cervical spine may be beneficial for individuals who suffer from CEH, although heterogeneity of the studies makes it difficult to generalize the findings.

## Introduction

It has been estimated that 4.1% of the population may experience cervicogenic headache (CEH) (1). CEH is one of the more common types of headache and may account for 0.4–15% of the headache population (2) and up to 15–20% of all chronic and recurrent headaches (3). Women have been reported to be affected four times more frequently than men (4), although some research about prevalence between the sexes is contradictory (1). Patients who have sustained concussion (5) or whiplash injuries (6) with resulting neck pain and limitation of movement can also develop CEH.

Cervicogenic headache was originally described as a unique disorder in 1983 and differentiated from other forms of headaches, such as migraine, that may present with some common symptoms (7). The International Headache Society (IHS) issued its initial International Classification of Headache Disorders (ICHD) in 1988 (8) and published revised editions in 2004 (9) and in 2013 (10). The current ICHD III beta version classifies CEH as a secondary headache arising from musculoskeletal disorders in the cervical spine and is frequently accompanied by neck pain (10). Additionally, the Cervicogenic Headache International Study Group (CHISG) has also developed a list of clinically relevant diagnostic criteria that include pain with neck movement or sustained improper positioning, restricted cervical range of motion (ROM), and ipsilateral shoulder and arm pain (11).

The symptoms of CEH many arise from any of the components of the cervical spine, including vertebrae, disks, or soft tissue (6), though some research has shown that CEH pain most commonly arises from the second and third cervical spine (C2/3) facet joints, followed by C5/6 facet joints (12). Furthermore, CEH symptoms can be successfully managed at the second and third cervical rami (C2/3) with cervical rami blocks (13). The afferent fibers of the trigeminal nerve and the upper three cervical nerves converge on second-order sensory neurons at the dorsal horn of the upper cervical spinal cord (14). This convergence is the anatomical basis for the clinical observation that patients with CEH often present with headache at both cervical and trigeminal dermatomes (15). Upper cervical spine mobility restriction (hypomobility), cervical pain, and muscle tightness are clinical findings associated with CEH during physical examination (16).

Because CEH is a secondary type of headache, it is important for physical therapists to perform a manual examination of the vertebral segments in order to determine the primary cause of the headache from the dysfunctional cervical spine segment (17). Manual examinations may include passive physiological intervertebral motion as well as passive accessory intervertebral motion such as posteroanterior pressures. Motion limitation and symptoms, such as pain, headache reproduction, and stiffness, indicate the most dysfunctional segment at the time of the manual assessment (14, 18). Due to the various anatomical and physiological dysfunctions, cervical mobilization and manipulation (manipulative therapy) are the frequently used treatments for patients diagnosed with CEH, although the evidence surrounding the efficacy of these practices is conflicting.

Many studies on the short-term effectiveness and manual therapy to the cervical spine (mobilization and manipulative therapy) have found it beneficial in reducing headache pain or disability (19–25), intensity (19–22, 24, 26), frequency (19, 26, 27), and duration (19, 22–27). Therapeutic effects for CEH patients were also found in terms of improvement in neck pain and disability (17, 20). Current research suggests that afferent input induced by manual therapy may stimulate neural inhibitory pathways in the spinal cord and can also activate descending inhibitory pathways in the lateral periaqueductal gray area of the midbrain (13). Jull et al. concluded that manual therapy is effective in managing CEH, and the effects of this study were maintained at a long-term follow-up. Thus, patients with CEH could benefit from manual therapy techniques, including spinal manipulative therapy (SMT) (28). If cervical manipulation is effective in reducing pain intensity and headache frequency in this patient population, this could be a beneficial treatment for physical therapists to incorporate into their interventions. However, evidence regarding the efficacy of this practice is conflicting (29). Therefore, we conducted a review to determine the effectiveness of manual therapy on the cervical spine, including SMT.

## Methods

We conducted literature searches to identify studies on the effect of cervical spine mobilization and manipulation in reducing pain and frequency of headaches in patients with CEHs in Cochrane, Embase, PubMed, PEDro, Clinicaltrials.gov, and Google Scholar. The following search terms were used: cervicogenic headache AND manipulation, cervicogenic headache AND low amplitude high velocity, cervicogenic headache AND adjustment, cervicogenic headache AND therapy, and cervicogenic headache AND treatment.

## Selection of Studies

Studies meeting the following criteria were considered for review: (1) randomized controlled trial (RCT); the study contained at least two separate groups that were randomly assigned either to a cervical spine mobilization or manipulation or a group that served as a comparison control; (2) subjects must have had a diagnosis of CEH; (3) the treatment group received spinal mobilization or manipulation, while the control group received another physical therapy intervention; and (4) the study included headache pain and frequency for outcome measurements. Studies were excluded for the following reasons: (1) subjects were diagnosed with another form of headache (migraine or tension headache), (2) studies that were case reports, (3) studies not appearing in peer-reviewed journals, and (4) studies that were not published in English.

A total of 66 relevant studies were originally identified through the database searches. Of those, 42 were excluded on the basis of the review of the title and abstract. A total of 25 studies were fully evaluated *via* a careful review of the text. On the basis of inclusion and exclusion criteria, 15 studies were excluded, leaving a total of 10 studies to be included in the review.

## Study Characteristics

The 10 included studies all investigated the effect of mobilization or manipulation of the cervical spine in reducing pain and headache in patients with CEH. The characteristics of those studies are summarized in Table 1. In three studies, the control group received some form of modalities ranging from low-level laser to moist heat. Other control groups took part in a low load endurance exercise program, received some form of massage ranging from deep friction and light soft tissue massage, or received an alternate PT intervention. Four control groups received a placebo (sham) intervention.

**Table 1:** **Description of included studies**.

	Subjects	Experimental intervention	Control or comparison intervention	Time of follow-up	Outcome measures
Borusiak et al. (29)	*N* = 52; *n* = 26 spinal manipulative therapy (SMT), *n* = 26 placebo	HVLA treatment session after 2-month baseline documentation	Light touch of cervical spine after 2-month baseline documentation	2 months	Percentage of days with headache, duration of headache, school absence due to headache, analgesic consumption, and headache intensity
Dunning et al. (19)	*N* = 110; *n* = 58 manipulation, *n* = 52 mobilization	6–8 SMT sessions for 4 weeks	Mobilization (6–8 sessions) and exercise for 4 weeks	1 week, 4 weeks, and 3 months	Numeric pain rating scale, headache frequency, duration, and disability using NDI
Haas et al. (20)	*N* = 23 completed	1, 3, and 4 visits for 3 weeks – HVLA	Heat and soft tissue therapy. Modification of ADL and rehab exercises	12 weeks	Disability scales and HA scale
Haas et al. (21)	*N* = 80; *n* = 40 experimental, *n* = 40 control	SMT – HVLA spinal manipulation of the CS and TS; 8 or 16 treatments	5 min of moist heat, 5 min of light massage; 8 or 16 treatments	4, 8, 16, and 20 weeks by phone; 12 and 24 weeks *via* mail	Neck pain and disability using the 100-point modified Von Korpf scale. Number of CEH and other HA, medications
Hall et al. (25)	*N* = 32; *n* = 16 SNAG, *n* = 16 placebo	Subjects taught C1-C2 self-SNAG mobilization using cervical strap; 2 repetitions 2× daily for 12 months	Sham mobilization with self-SNAG cervical strap; 2 repetitions 2× daily for 12 months	4 weeks and 12 months	ROM (flexion rotation test), long-term self-reported headache symptoms (VAS and questionnaire)
Jull et al. (17)	*N* = 200; *n* = 49, 51, and 52 experimental; *n* = 48 control	MT – low velocity cervical joint mobilization and high velocity manipulation techniques to the cervical spine; MT and Thera Ex combined; Thera Ex	Thera Ex – low load endurance exercise to train muscle control of the cervicoscapular region	3, 6, and 12 months	Northwick Park neck pain questionnaire, changes in HA frequency, intensity (VAS), and duration (average number of hours that HA lasted in the past week)
			Control group – received no PT intervention		
Khan et al. (23)	*N* = 60; *n* = 30 SNAG, *n* = 30 posterior anterior vertebral mobilization (PAVM)	SNAG treatment and ice on cervical spine for 6 sessions over 6 weeks	PAVM treatment and ice on cervical spine for 6 sessions over 6 weeks	Posttest at 6 weeks	Pain (VAS) and disability using NDI
Nilsson et al. (24)	*N* = 53; *n* = 28 experimental, *n* = 25 control	Low-amplitude cervical manipulation 2×/week for 3 weeks	Low-level laser in the upper cervical region and deep friction massage	5 weeks	Change in analgesic use per day from week 1 to week 5, HA intensity per episode, and number in HA hours/day
Shin and Lee (22)	*N* = 40; *n* = 20 SNAG, *n* = 20 control	Mulligan SNAG treatment, 20 min 3×/week for 4 weeks	Placebo (contact only); 12× in 4 weeks	Pre- and post-assessment; no long-term follow-up	VAS for pain, NDI, and headache duration
Youssef and Shanb (26)	*N* = 35; *n* = 18 mobilization, *n* = massage	HVLA cervical spine manipulation; AROM, strengthening, endurance exercises	Massage; active range of motion (AROM), strengthening, and endurance exercises	Pre- and post-assessment; no long-term follow-up	HA pain, intensity, duration, NDI, and AROM

*ADL, activities of daily living; AROM, active range of motion; HVLA, high velocity, low amplitude; NDI, neck disability index; SNAG, sustained natural apophyseal glide; VAS, visual analog scale*.

All of the studies reported some form of an outcome measure for the HA intensity, frequency, and duration. These measures were reported prior to the treatment, immediately posttreatment, and/or at follow-up at several weeks posttreatment. Long-term follow-ups were conducted in three of the studies. Hall et al. (25) and Jull et al. (17) made follow-up assessments 12 months post-intervention, and Haas et al. (21) followed up *via* mail at 24 months post-intervention.

A total of 685 subjects were used in the 10 studies. The subject gender was a mixture of males and females in all studies except for Shin and Lee (22), which focused on a patient population of women with CEH. Borusiak et al. (29) studied a sample of children and adolescents with CEH. The frequency and duration of the spinal mobilization and manipulation greatly varied among studies, ranging from one mobilization or manipulation per week to several self-administered manipulations or mobilizations per day.

## Results

Seven studies examined how the effects of SMT compared to an alternate intervention or a placebo. Of these seven, six studies found statistically significant improvements in symptoms for participants in the manipulation group as compared to controls. Several of the studies looked at pain intensity, headache frequency, duration, disability, or ROM. Haas et al. (21) found that SMT was more effective at reducing pain intensity and disability when compared to light massage (*p* < 0.05), and these effects were larger after 16 sessions than after 8. They also discovered that the mean number of CEHs was reduced for the SMT group, and this improvement was maintained at a 24-week follow-up. Nilsson et al. (24) found that participants in the SMT group had a greater reduction in pain and analgesic use (*p* = 0.04), number of headache hours per day (*p* = 0.03), and headache intensity (*p* = 0.03) compared to participants receiving low-level laser and deep friction cervicothoracic massage. Youssef and Shanb (26) also compared a mobilization and a massage intervention for participants with CEH and found that mobilization was more effective at reducing pain intensity, frequency, and duration (*p* < 0.05) than massage. Both groups had improvements for the outcome of disability, and there was not a significant difference between the groups for this outcome measure. This is contradictory to Haas et al.’s finding that disability was reduced more with SMT than massage, although these studies used different disability indexes.

Dunning et al. (19) was the only study included that compared the efficacy of manipulation to mobilization techniques. In this study, participants were randomized into either a manipulation intervention group or a combined mobilization and exercise group. The treatments and exercise program lasted 4 weeks, and participants received six to eight sessions of manipulation or mobilization. The techniques used targeted both upper cervical and upper thoracic spine, and the specific segments were selected based on the patients’ symptoms and the physical examination. The findings of this study indicated that manipulation was more effective at reducing CEH intensity and disability at 1 week, 4 weeks, and 3 months (*p* < 0.001 for all). Additionally, the manipulation group experienced significantly reduced duration and frequency of headaches as well as perceiving greater improvement (*p* < 0.001 for all). These findings suggest that the high-velocity, low-amplitude (HVLA) manipulation was more effective at treating CEH than the slow rhythmic mobilization techniques used as an intervention.

Jull et al. (17) conducted a study on the effects of therapeutic exercise, manipulation, and the combined effects of therapeutic exercise and manipulation for individuals with CEH. After 7 weeks, all three groups had reduced means for headache frequency, intensity, and neck pain (*p* < 0.05 for all). The manipulation group and combined intervention group also had a significant reduction in duration at this time. After 12 months, all three groups had reduced means for frequency and intensity, and the combined intervention group had reduced headache duration and neck pain (*p* < 0.05 for all). The control group did not have any significant changes form baseline. These findings highlight the possible lasting benefits of combining therapeutic exercise with manipulation for patients with CEH.

Shin and Lee (22) and Hall et al. (25) studied the efficacy of a sustained natural apophyseal glide (SNAG) intervention compared to a placebo control group. Shin and Lee found that the SNAG intervention group had greater reductions in disability, intensity, and duration than the control group. This study was unique in that it was the only one that looked specifically at the efficacy of SMT on a sample of females with CEH. Hall et al. found greater improvements in ROM for participants in the SNAG group compared to the control subjects (*p* < 0.001) and a significant reduction in self-reported symptoms at both 4 weeks and 12 months for the SNAG intervention group than the control group.

One of the other two studies investigated dosage effects of SMT on CEH (20), and the other compared the effectiveness of two different forms of SMT for individuals with CEH (23). Haas et al. (20) looked at the relationship between treatment frequency and patient outcomes for subjects receiving one, three, or four treatments per week. After 4 weeks, subjects receiving four visits per week had significant reductions in headache pain and intensity compared to the subjects receiving one treatment per week; and after 12 weeks, subjects receiving three or four visits per week had reduced pain and intensity compared to the once-per-week treatment group (*p* < 0.05 for all). There were no differences between the subjects receiving three and four treatments per week. This suggests that there may be an optimal dosage effect for SMT intervention and that, to a certain extent, more frequent treatments may be related to more significant positive outcomes. Khan et al. (23) examined how a SNAG intervention compared to posterior anterior vertebral mobilization (PVAM) in treating CEH. Their research revealed that although both groups had improvements in neck disability index (NDI) and visual analog (VAS) scores, the SNAG treatment group was more effective for both NDI (*p* = 0.004) and VAS (*p* < 0.001). These findings indicate some mobilizations or manipulations may have greater efficacy than others in reducing CEH symptoms, though additional studies would be needed to draw a firm conclusion about what is most effective in the clinical setting.

Of the studies included in this review, only Borusiak et al. (29) did not find any statistically significant improvements for the manipulation group as compared to the placebo in a sample of children and adolescents. Since few studies have examined the effects of SMT in this population, further studies would be needed to draw any conclusions about the significance of these findings.

## Discussion

The manual therapy techniques for the experimental groups, which included spinal mobilizations and manipulation interventions, varied across the 10 included studies. Comparison interventions were also heterogeneous and ranged from light massage, laser, therapeutic exercises, alternate physical therapy interventions, and placebo treatments. Additionally, primary outcome measures varied and included patient diaries, the Numeric Pain Rating Scale, and Modified Von Korf pain and disability scale. Diaries recorded total number of days with headache symptoms, headache duration, analgesic consumption, headache intensity per episode, and the number of headache hours per day. The frequency and duration of spinal mobilization and manipulative therapy varied across studies from a single session to multiple sessions. Given such variability and lack of manual therapy standardization, it is difficult to draw a firm clinical significance. Taken independently, the findings of the studies suggest that manual therapy on the cervical spine is more effective than traditional physical therapy interventions or sham intervention in reducing pain intensity and frequency of headaches in this population. However, it is difficult to synthesize the findings and assign them clinical significance.

Additionally, since there is a temporal range in which the studies were conducted, participant CEH diagnosis was determined under different ICHD editions or CHISG diagnostic criteria. Thus, there may have been some variability in patient presentation and subject eligibility. Some research indicates that the ICHD classifications may be a work in progress, especially as many primary and secondary headaches are still poorly understood (30, 31). As research continues to expand the knowledge about the pathophysiology involved in headaches, such as CEH, the criteria for differential diagnosis will become clearer and, perhaps, there will be more cohesive evidence-based interventions used in treatment.

In spite of a growing body of evidence supporting cervical manipulation, further studies on this topic are needed to accurately assess the effectiveness of this technique for treating patients with CEH. The findings of most of the included studies indicate manipulation or mobilization as promising interventions. However, the high heterogeneity in treatments, possibly due to the small number of studies included, suggests that the results should be interpreted cautiously. There is a lack of standardization in SMT interventions and outcome measures in the literature. Further research is required to establish a strong evidence-based foundation for use of these interventions in CEH patients.

## Limitations

Our review has several limitations. Our searches were thorough but were limited to articles published in English. Thus, we cannot be sure that all relevant articles were identified. The total number of trials included in our review and the heterogeneity of the studies prevent us from concluding that manipulation of the cervical spine is an effective treatment for CEH.

## Author Contributions

Concept development (provided idea for research): JG, SA, KT, and KV; supervision (provided oversight, responsible for organization and implementation, and writing of the manuscript) and analysis/interpretation (responsible for statistical analysis, evaluation, and presentation of results): JG and RF; and design (planned methods to generate the results), data collection/processing (responsible for organization and data reporting), and writing (responsible for writing substantive part of the manuscript): JG, SA, KT, KV, and RF.

## Conflict of Interest Statement

The authors declare that the research was conducted in the absence of any commercial or financial relationships that could be construed as a potential conflict of interest.
